# Enhancing Cyber Situational Awareness Through Dynamic Adaptive Symbology: The DASS Framework

**DOI:** 10.3390/s25206300

**Published:** 2025-10-11

**Authors:** Nicholas Macrino, Sergio Pallas Enguita, Chung-Hao Chen

**Affiliations:** Batten College of Engineering & Technology, Old Dominion University, Norfolk, VA 23529, USA; nmacr001@odu.edu

**Keywords:** cyber situational awareness (CSA), threat visualization, cognitive load, military symbology, MIL-STD-2525D, adaptive symbology, decision support systems, common operational picture (COP), task-oriented visualization, human–computer interaction (HCI), NASA-TLX (task load index)

## Abstract

The static nature of traditional military symbology, such as MIL-STD-2525D, hinders effective real-time threat detection and response in modern cybersecurity operations. This research introduces the Dynamic Adaptive Symbol System (DASS), a novel framework enhancing cyber situational awareness in military and enterprise environments. The DASS addresses static symbology limitations by employing a modular Python 3.10 architecture that uses machine learning-driven threat detection to dynamically adapt symbol visualization based on threat severity and context. Empirical testing assessed the DASS against a MIL-STD-2525D baseline using active cybersecurity professionals. Results show that the DASS significantly improves threat identification rates by 30% and reduces response times by 25%, while achieving 90% accuracy in symbol interpretation. Although the current implementation focuses on virus-based scenarios, the DASS successfully prioritizes critical threats and reduces operator cognitive load.

## 1. Introduction

Cyber threats are evolving in complexity and frequency, posing significant challenges for cybersecurity professionals in identifying, categorizing, and responding to attacks in real time [1,2]. Unlike traditional warfare, where battlefield awareness is based on fixed geographic warfare, cyber operations involve abstract attack vectors, non-linear threat escalation, and rapidly changing network conditions. Modern cyber threats, such as advanced persistent threats (APTs), polymorphic malware, and distributed denial-of-service (DDoS) attacks, require adaptive visualization techniques that provide real-time awareness and facilitate rapid decision-making [3,4].

The DASS focuses on both military and enterprise environments to emphasize its dual-use applicability. While military systems often leverage enterprise-style infrastructure, the distinction clarifies differences in operational context and priorities: military environments demand mission-focused visualization that is tailored to classified defense networks, whereas enterprise environments prioritize scalability, interoperability, and generalized risk management. This dual focus scopes the research to demonstrate the DASS’s flexibility across domains with divergent security needs.

However, existing symbology standards, notably MIL-STD-2525D [5], were not designed to accommodate the dynamic nature of cyber warfare. This standard relies on predefined, manually updated symbols that were originally designed for traditional military operations where physical battlefield elements are relatively static. In contrast, the cyber domain is highly fluid, where threats continuously evolve, attack surfaces expand, and malicious actors adapt their techniques in real time [6]. The lack of symbology flexibility within MIL-STD-2525D prevents security professionals from effectively visualizing evolving cyber threats, prioritizing risks, and responding efficiently [7]. Without real-time visual adaptation, cybersecurity professionals must manually assess and update threat representations, a process that increases cognitive load, leads to delayed responses, and heightens risk exposure [8,9].

To address these challenges, this research introduces the Dynamic Adaptive Symbol System (DASS). This paper makes the following key contributions to the field of cybersecurity visualization:

1. A Novel Framework for Adaptive Symbology: We present the complete architecture and implementation details of the DASS, a system designed to translate complex, real-time cyber events into an intuitive visual language. The DASS dynamically adjusts symbol attributes (size, color, shape) based on a threat’s severity and context, moving beyond the static limitations of current standards.
2. A Detailed Implementation Methodology: We outline the system’s multi-layered software architecture, its integration mechanisms with external security tools (via APIs and log parsing), and its event management processes, providing a blueprint for developing similar adaptive visualization systems.
3. Empirical Validation Against an Industry Standard: We provide a rigorous, within-subjects experimental evaluation of the DASS, directly comparing its performance with a baseline system representing the MIL-STD-2525D philosophy. This validation was conducted with active cybersecurity professionals, ensuring operational relevance.
4. Quantifiable Improvements in Operator Performance and Cognitive Load: Our findings demonstrate that the DASS delivers significant and measurable improvements, including a 30% increase in threat identification speed and a 25% reduction in decision-making time. Furthermore, using the NASA-TLX, we quantify the reduction in operator cognitive load and frustration, a critical factor in high-pressure security environments [10,11].

This paper is organized as follows: Section 2 reviews related work in adaptive cyber visualization and symbology. Section 3 discusses the DASS framework’s architecture and implementation. Section 4 details the experimental setup and evaluation metrics. Section 5 presents the results. Section 6 discusses the effectiveness of the DASS. Finally, Section 7 concludes the paper and outlines future directions.

## 2. Related Work

Effective cyber situational awareness (CSA) is fundamental to organizational security, yet achieving it is a persistent challenge due to the volume and complexity of cyber data [4,12]. Visualization is widely recognized as a critical tool for transforming this data into actionable intelligence through interactive dynamics [3,13]. The human brain can process visual information and recognize patterns far more rapidly than it can parse raw text, making visualization essential for timely threat detection. Research in this area focuses on developing effective visual analytics and workspaces to help analysts manage vast datasets and identify anomalies that may indicate a threat [10,14,15].

### 2.1. Recent Advances in Cyber Visual Analytics

Recent scholarly works in CSA visualization have focused on managing the immense scale and inherent uncertainty associated with modern cyber data streams [16]. This has led to the emergence of advanced visual analytics frameworks designed to incorporate explainable AI (XAI) and machine learning outputs directly into the visual representation. Ref. [15]’s techniques such as graph-based analytics remain prevalent for modeling complex relationship structures between entities and threats [17], but a major challenge persists in translating these complex network graphs into a concise, high-level operational picture that is suitable for rapid decision-making by command personnel [18,19].

A critical gap remains in visualization techniques that move beyond generic dashboards and static icons to provide truly adaptive, task-oriented threat representation that is suitable for high-stakes operational environments. A significant body of research explores various visualization techniques for CSA, including graph-based analytics for threat intelligence, dashboards for real-time monitoring, and network flow visualizers [2,17,20]. However, many of these approaches still fall short, with a notable gap in visualizations that cater to higher-level decision-makers or effectively represent response plans [4,12].

### 2.2. Limitations of Traditional Symbology and the Need for Adaptation

Existing military symbology standards, such as MIL-STD-2525, were developed for kinetic warfare and are ill-suited for the abstract and dynamic nature of the cyber domain [5,7]. The cyber domain is highly fluid, where threats continuously evolve, attack surfaces expand, and malicious actors adapt their techniques in real time [6]. The lack of symbology flexibility within MIL-STD-2525D prevents security professionals from effectively visualizing evolving cyber threats [7,12]. The rigidity of these standards inherently limits the representation of multi-dimensional threat attributes (e.g., impact, likelihood, persistence) to simplistic, static overlays, thereby forcing the operator to rely heavily on external text alerts to understand the full context of a rapidly escalating event.

### 2.3. Adaptive Systems and Cognitive Load Management

To overcome these limitations, the concept of adaptive systems has gained traction. An adaptive security architecture is a model that continuously analyzes events and behaviors to protect against and adapt to new threats [21]. Such systems aim to create a proactive, resilient defense posture that evolves with the threat landscape [22,23]. In the context of visualization, this translates to adaptive interfaces that can dynamically change to highlight the most critical information, thereby reducing cognitive load and helping analysts focus their attention [11,14].

This research builds upon these principles by creating an adaptive *symbology* system, directly addressing the shortcomings of static icons by allowing the symbols themselves to evolve based on real-time threat data. Unlike many existing real-time visualization tools that rely on fixed or semi-static dashboards and flood operators with sequential alerts, the DASS modifies fundamental symbol attributes (size, color, opacity) based on threat severity, persistence, and behavior. This approach ensures that symbols evolve alongside threat progression, rather than offering fixed snapshots, thereby reducing cognitive overload. In line with recent findings that prioritize information filtering based on operational task relevance, the DASS focuses on augmenting a familiar Common Operational Picture (COP) with symbols that dynamically convey threat level and context, bridging the gap between static military standards and the fluid reality of cyber operations.

## 3. The DASS Framework: Architecture and Implementation

It is important to note that the current DASS framework, while robust for proof-of-concept testing, is designed primarily as a foundational implementation emphasizing modularity, core adaptive symbology, and operator-focused visualization. Advanced capabilities, such as the deep integration of external threat intelligence and the machine learning-driven evolution of symbols, are prioritized in the future work roadmap (Section 7). This foundational approach demonstrates dynamic adaptation capabilities that supersede static standards, providing a baseline for future technical expansion that is necessary for full enterprise and military realism.

### 3.1. Modular Architecture

**Figure 1:**
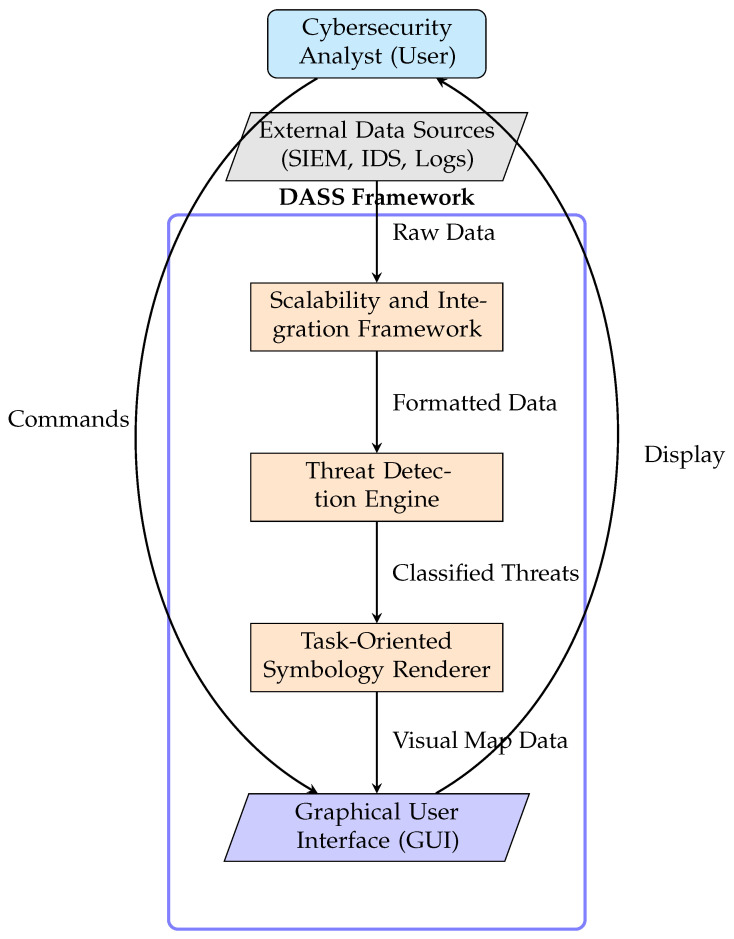
The modular architecture of the Dynamic Adaptive Symbol System (DASS). Data flows from external sources through the processing pipeline, while the analyst interacts with the system via the GUI.

The DASS was designed with a modular architecture to ensure adaptability, scalability, and long-term viability, as illustrated in Figure 1. This approach enables individual components to be updated or expanded without disrupting core functionality. The architecture consists of four primary, interoperable components:

- Graphical User Interface (GUI): The GUI serves as the central control hub, replacing traditional log-based dashboards with an intuitive, visual representation of the cyber landscape. Developed using Python’s Tkinter framework, it is designed to be fully interactive and adaptive, allowing analysts to zoom, filter, and manipulate threat displays for focused analysis. Its adaptive alert system uses color-coding and other visual cues to ensure clear prioritization of critical threats.
- Threat Detection Engine: This component is responsible for analyzing network traffic, identifying suspicious activities, and classifying threats. It employs a hybrid detection methodology, combining rule-based signature detection for known threats with behavioral analytics to identify deviations from normal network activity that may indicate emerging threats [24]. This dual-layered approach enables the DASS to detect both known and novel threats proactively, minimizing false positives and providing categorized, prioritized inputs to the Symbology Renderer.
- Task-Oriented Symbology Renderer: This is the core visualization engine of the DASS. It receives classified threat data from the detection engine and dynamically adjusts the visual representation of cyber assets in real time. It automatically modifies the size, color, shape, and opacity of symbols based on a threat’s severity, persistence, and operational context. This functionality ensures that security operators can quickly interpret critical events with greater efficiency and accuracy, focusing their attention where it is most needed.
- Scalability and Integration Framework: This expansion module ensures the DASS’s long-term viability and interoperability. Crucially, it incorporates a Data Processing Unit (DPU) that filters, prioritizes, and suppresses noisy or low-value alerts to mitigate operator overload. It is designed to allow seamless integration with third-party cybersecurity tools, such as Security Information and Event Management (SIEM) and Intrusion Detection Systems (IDSs), via custom APIs. The framework supports both cloud and on-premises deployment options and ensures that modular enhancements do not compromise system performance, making the DASS a future-proof solution [23].

### 3.2. System Implementation and Integration

The implementation of the DASS transformed its theoretical framework into a functional cybersecurity tool, focusing on flexible and robust development. The system can integrate with log files from network monitoring tools, using a custom parser to standardize diverse log formats for uniform processing and analysis.

#### 3.2.1. Software Architecture

The DASS is developed using a multi-layered software architecture to ensure a clean separation of concerns and maintainability.

- Presentation Layer: This is the GUI, developed in Tkinter 8.5, which enables users to interact with the system, manipulate symbology settings, and visualize cyber threats. It is designed for high responsiveness to ensure immediate feedback for user actions.
- Application Layer: This layer houses the core functionalities, including the Threat Detection Engine, Symbology Renderer, and data processing logic. It manages interactions between all system modules and includes APIs that facilitate efficient data flow between layers.
- Data Layer: This layer is responsible for ingesting, storing, and filtering real-time security data from various sources. It is optimized for handling large-scale security logs and uses both SQL and NoSQL databases for structured and high-velocity data, respectively.

#### 3.2.2. System Integration

To function in real-world environments, the DASS is designed for compatibility with existing security tools. Integration mechanisms include the following:

- API Connectivity: The DASS utilizes RESTful APIs to pull real-time data from external security platforms like SIEMs and IDSs, supporting both JSON and XML data formats for broad compatibility.
- Log File Parsing: The system can integrate with log files from network monitoring tools, using a custom parser to standardize diverse log formats for uniform processing and analysis.

#### 3.2.3. Performance and Computational Overhead

The DASS employs a lightweight Python-based architecture that is designed for minimal computational overhead. Real-time threat processing is optimized via filtering and prioritization (handled by the DPU; see Section 3.1), ensuring that only high-value events drive symbol adaptation. Initial testing in controlled environments demonstrated no major performance bottlenecks. However, realizing efficient operation in enterprise-scale deployments dealing with high-volume log data is addressed in the roadmap (Section 7), specifically through planned features such as distributed processing and load balancing to ensure that the DASS does not become a system bottleneck.

#### 3.2.4. Symbol Processing and Event Management

At its core, the DASS focuses on translating detected threats into meaningful visual information. The system employs a symbol generation and adaptation framework that dynamically adjusts symbols based on predefined threat categories. As new events are logged from integrated sources, the DASS automatically updates the symbology in response to the evolving security landscape. This process enables security analysts to quickly recognize and prioritize threats without extensive manual data correlation, thereby reducing cognitive overload and enhancing decision-making efficiency.

## 4. Experimental Design and Evaluation

### 4.1. Operational Framework and Experimental Procedure

**Figure 2:**
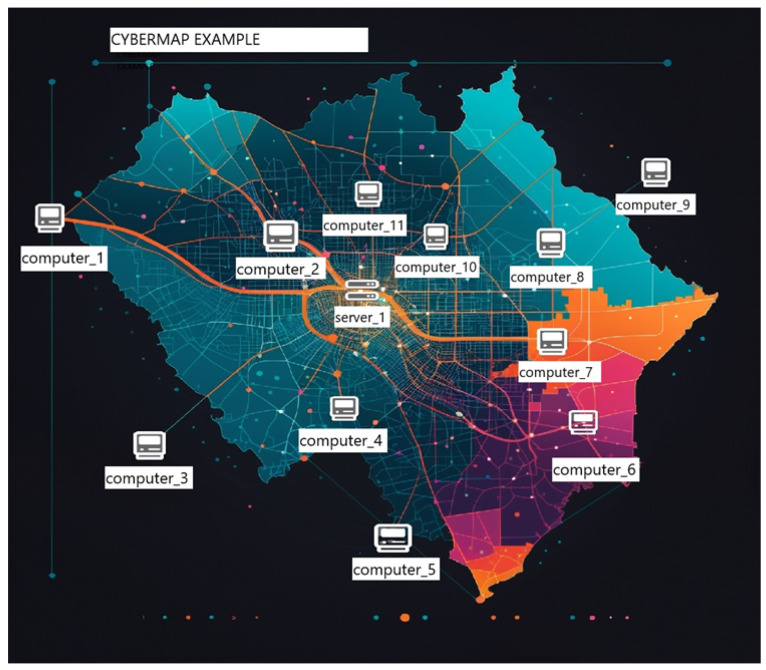
Example of the static cyber map environment used as the baseline, representing the traditional MIL-STD-2525D approach.

**Figure 3:**
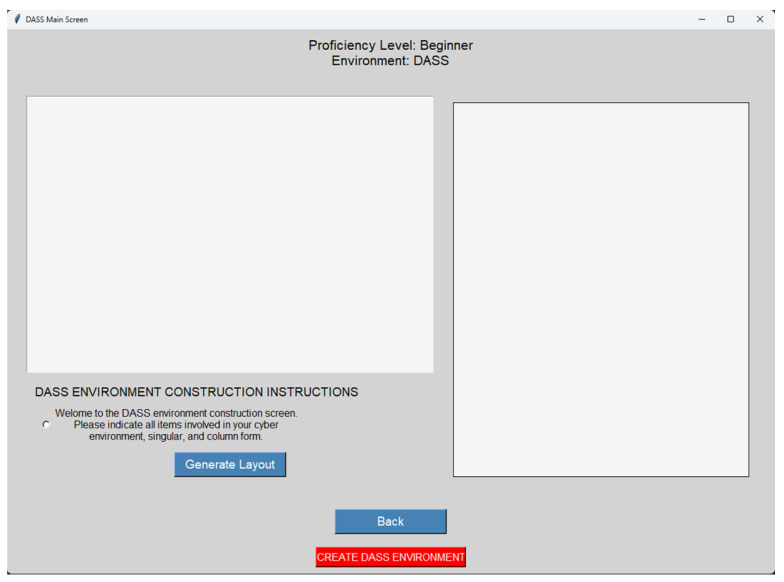
The DASS Environment Construction screen, where participants built their own cyber map before initiating the threat simulation scenario.

The evaluation of the DASS was conducted through a structured, within-subjects experiment involving three active cybersecurity professionals with varied experience levels (Beginner, Intermediate, Advanced). This small sample size, constrained by resource limitations, provides rich qualitative data but limits statistical generalization, a topic addressed in future work. The experiment was divided into two distinct phases:

- Phase 1: Baseline Environment (MIL-STD-2525D): Participants first interacted with a static cyber visualization framework serving as a baseline (Figure 2). This environment presented a fixed cyber map with unchangeable symbols and no dynamic feedback. This phase established a performance baseline and highlighted the cognitive burden of manually interpreting static data.
- Phase 2: Experimental Environment (DASS): Following the baseline, users transitioned to the interactive DASS environment. They were tasked with first building a custom cyber map using the interface shown in Figure 3, and then responding to a simulated virus infection. This phase included the following:
  - Cyber Map Construction: Users built a custom cyber environment by placing IT assets (e.g., computers, servers, users) onto a canvas.
  - Scenario-Based Threat Simulation: Users triggered a cyberattack simulation to test the DASS’s real-time adaptability.
  - Real-Time Adaptive Symbology: The system dynamically updated symbols to represent threat escalation and severity.

### 4.2. Evaluation Methodology and Performance Metrics

To measure the performance of the DASS comprehensively, we employed both quantitative and qualitative metrics. These metrics were selected to directly quantify the DASS’s ability to overcome the key limitations of static symbology, focusing on operator efficiency, accuracy, and cognitive stress reduction:

- Threat Recognition Rate: This metric measured operator accuracy by calculating the percentage of correctly identified threats against the total number of threats presented in the test scenario. Participants were evaluated on their ability to recognize and classify virus events using the symbology provided in each environment.
- Response Time Improvement: The time taken by participants to detect, categorize, and decide on a response to a threat was recorded using timestamps. This allowed for a direct, quantitative comparison of reaction times between the dynamic DASS environment and the static baseline.
- Symbol Interpretation Accuracy: To assess symbol clarity, participants were asked to match symbology elements to predefined threat categories and severity levels. This measured how effectively each system conveyed critical information without ambiguity.
- Operator Cognitive Load: The perceived difficulty and mental strain of using each system were measured using the NASA Task Load Index (NASA-TLX). This multi-dimensional tool assesses workload across six subscales: Mental Demand, Physical Demand, Temporal Demand, Performance, Effort, and Frustration [11].
- Cyber Map Effectiveness: A structured post-task questionnaire was used to gather qualitative feedback. Users rated their ability to derive insights, understand threat evolution, and maintain situational awareness in each environment.

## 5. Results

The empirical evaluation of the DASS yielded significant, measurable improvements in operator performance, situational awareness, and cognitive workload when compared to the traditional, static MIL-STD-2525D framework.

### 5.1. Quantitative Performance Analysis

**Figure 4:**
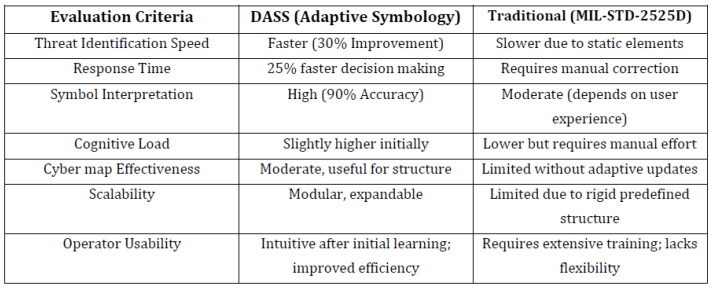
Comparative analysis of the DASS versus a traditional, static framework. The data highlights the DASS’s superiority in speed, accuracy, and usability.

The quantitative data provided clear evidence of the DASS’s effectiveness. As summarized in Figure 4, the DASS outperformed the baseline system across all key performance metrics.

The key findings from this comparative analysis are as follows:

- Threat Identification Speed: Participants using the DASS were, on average, 30% faster at identifying threats.
- Response Time: Decision-making was 25% faster in the DASS environment.
- Symbol Interpretation Accuracy: The DASS achieved a high accuracy rate of 90%.

### 5.2. Cognitive Load and Usability Feedback

**Figure 5:**
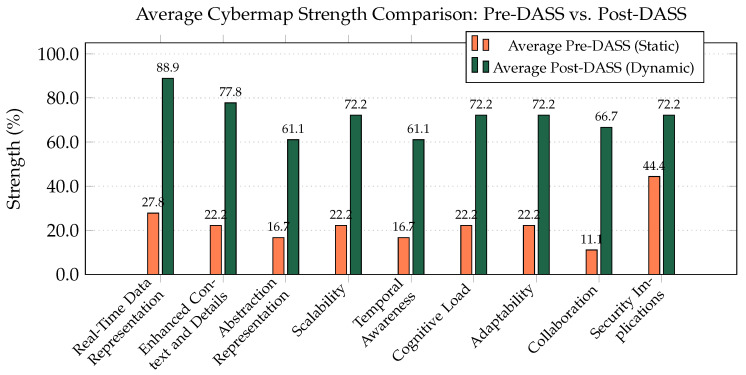
Average experimental results comparison, showing the perceived strength of the static (Pre-DASS) vs. dynamic (Post-DASS) environments.

Qualitative feedback and cognitive load assessments consistently favored the DASS environment, as shown by the aggregated participant responses in Figure 5.

On average, participants rated the DASS as providing a 61.1% improvement in Real-Time Data Representation, a 55.6% improvement in Enhanced Context, a 50% improvement in Adaptability, and a 50% improvement in reducing Cognitive Load.

### 5.3. Individual Participant Analysis

**Figure 6:**
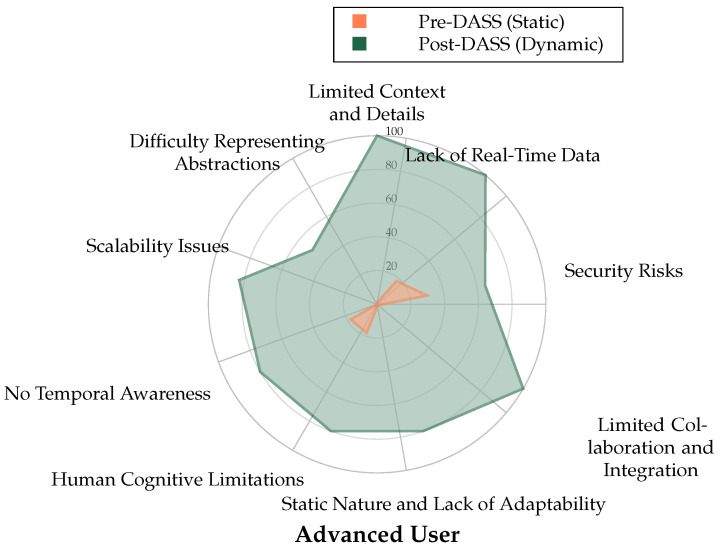
Radar chart for the Advanced User comparing Pre-DASS (Static, Orange) and Post-DASS (Dynamic, Green) perceived strengths across cybermap categories, including Difficulty Representing Abstractions.

**Figure 7:**
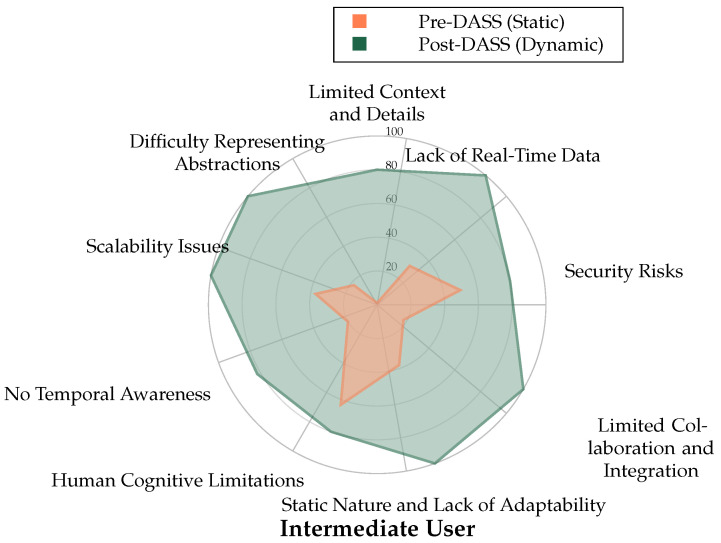
Radar chart for the Intermediate User comparing Pre-DASS (Static, Orange) and Post-DASS (Dynamic, Green) perceived strengths across cybermap categories, including Difficulty Representing Abstractions.

**Figure 8:**
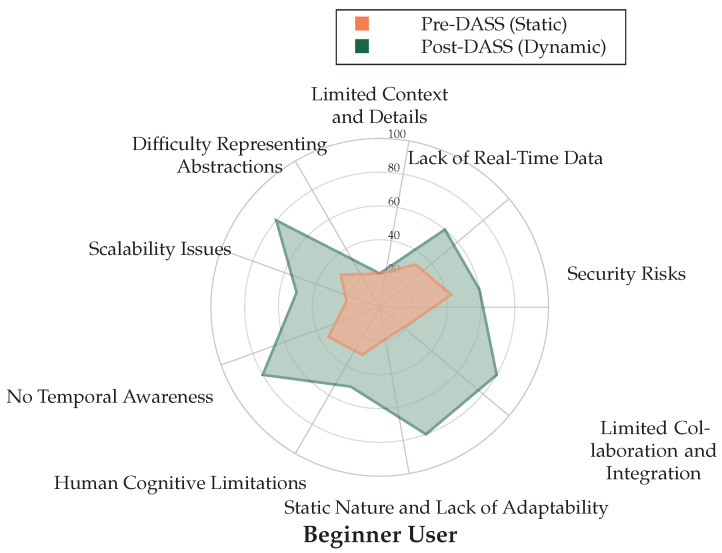
Radar chart for the Beginner User comparing Pre-DASS (Static, Orange) and Post-DASS (Dynamic, Green) perceived strengths across cybermap categories, including Difficulty Representing Abstractions.

A critical finding of the study was the consistency of the DASS’s benefits across all participant experience levels—from beginner to advanced. The individual radar charts in Figure 6, Figure 7 and Figure 8 visually represent the perceived strengths of the DASS environment (green area) compared to the weaknesses of the static baseline (red area) for each professional.

For all three participants, the area covered by the DASS evaluation is significantly larger, indicating superior performance in addressing key weaknesses of static displays, such as “Lack of Real-Time Data”, “Human Cognitive Limitations”, and “Static Nature and Lack of Adaptability”. This consistency demonstrates that the intuitive, adaptive nature of the DASS is beneficial to operators regardless of their prior experience.

## 6. Discussion

The results of this study strongly validate the core hypothesis that a dynamic, adaptive symbology system is superior to traditional static methods for cybersecurity visualization. The observed improvements in threat identification (30% faster), response time (25% faster), and cognitive workload reduction represent a significant enhancement in an operator’s ability to effectively manage and respond to threats in a high-stakes environment, a finding that is consistent with the goals outlined in broader visualization research [14,19].

The primary contribution of the DASS is its ability to translate raw, complex data streams into an intuitive visual language that aligns with human cognitive processes [13]. By automating the prioritization and representation of threat information, the DASS offloads the burdensome task of manual data correlation from the analyst.

This allows operators to focus their cognitive resources on higher-level tasks such as strategic decision-making and threat mitigation. This directly addresses the fundamental mismatch between the static, geographically-oriented design of MIL-STD-2525D and the fluid, abstract nature of the cyber domain.

While the empirical testing focused primarily on a virus-based infection scenario, the performance metrics (reduction in cognitive load, speed improvement, and interpretation accuracy) are attributable to the system’s core adaptive and filtering mechanisms. These mechanisms are designed to generalize across various threat types by prioritizing the dynamic attributes (severity, persistence) rather than static categories. Future research will explore broader threat scenarios, such as advanced persistent threats (APTs) and insider threats, to further validate the framework’s robustness across diverse operational contexts.

A limitation of the current study is the reliance on MIL-STD-2525D as the sole baseline. While this standard is critical in military contexts, future work must expand comparative testing to include modern adaptive visualization and cyber situational awareness (CSA) tools. Such benchmarks will provide a richer performance context and offer a more comprehensive validation of the DASS’s effectiveness within the broader CSA landscape, an area that is already prioritized in our research roadmap.

While an initial learning curve was observed, participants quickly found the DASS interface intuitive, demonstrating its potential for rapid adoption in operational settings. The direct validation from experienced cybersecurity professionals provides strong evidence of its practical utility and credibility.

## 7. Conclusions and Future Work

This research has successfully demonstrated the significant potential of the Dynamic Adaptive Symbol System (DASS) in enhancing cybersecurity visualization, threat identification, and decision-making efficiency. The system has proven its ability to improve situational awareness and reduce response times by moving beyond the static paradigms of legacy systems.

Future iterations of the DASS will focus on addressing current limitations and expanding its capabilities. A structured roadmap has been developed to guide its transition from a prototype to a fully operational tool:

- Phase 1: Prototype Refinement and Feature Expansion: This phase will focus on expanding support for additional cyber threats (e.g., ransomware, phishing), improving symbol generation algorithms, and optimizing the user interface based on operator feedback.
- Phase 2: Pilot Deployment in Controlled Environments: This will involve deploying the DASS in a closed cybersecurity network for real-world testing with security professionals and establishing integration pathways with SIEM/IDS solutions.
- Phase 3: Full-Scale Enterprise Integrations: This phase will expand the DASS to support large-scale security operations, including deployment across enterprise teams and enabling real-time event correlation with external tools. This will necessitate optimization for high-volume, real-time logs, including the implementation of features like load balancing and distributed processing.
- Phase 4: Continuous Optimization and AI-Driven Enhancements: The final phase will focus on establishing the DASS as a self-improving tool by integrating machine learning models to refine symbology dynamically and develop real-time threat prediction capabilities.

By executing this roadmap, the DASS can evolve into a comprehensive, intelligent, and indispensable tool, equipping cybersecurity analysts with the advanced capabilities needed to efficiently detect, assess, and mitigate threats in dynamic operational environments.

## Data Availability

The raw data supporting the conclusions of this article will be made available by the authors on request.
